# Evaluation of immunohistochemical and gene expression of Janus kinase 1 and Janus kinase 3 in the skin of different clinical types of mycosis fungoides patients – Part 1^☆^

**DOI:** 10.1016/j.abd.2026.501299

**Published:** 2026-03-09

**Authors:** Heba Saed El-Amawy, Basma Mourad Mohamed Ali, Mohamed Moustafa Shareef, Lamia Elgarhy

**Affiliations:** a Department of Dermatology and Venereology, Faculty of Medicine, Tanta University, Tanta, Egypt; b Department of Pathology, Faculty of Medicine, Tanta University, Tanta, Egypt

**Keywords:** Cutaneous T-cell lymphoma, Immunohistochemistry, Janus kinases, Janus kinase-1, Janus kinase-3, Mycosis fungoides

## Abstract

**Background:**

Mycosis fungoides is the commonest type of cutaneous T-Cell lymphoma. Janus kinases are intracellular tyrosine kinases that have recently been proven to have a crucial role in the pathogenesis and progression of several dermatological and malignant diseases.

**Objective:**

The aim of this study was to investigate the immunohistochemical expression of Janus Kinase-1 (JAK1) and Janus Kinase-3 (JAK3) in the skin of mycosis fungoides .

**Methods:**

The current study included 46 patients with early-stage MF, and 7 patients with late-stage MF, and 53 control samples. Immunohistochemical staining using JAK1 and JAK3 monoclonal antibodies was done in the skin specimens obtained from patients and controls. The evaluation of JAK1 and JAK3 gene expression using RT-PCR will be included in part 2 of the study.

**Results:**

JAK1 immunohistochemical expression was nuclear, and JAK3 was cytoplasmic in MF patients. Immunoreactivity scores for JAK1 and JAK3 in MF patients were significantly increased compared to healthy controls (p < 0.001 and < 0.001, respectively). JAK1 and JAK3 immunoreactivity scores were significantly higher in the tumor than in the patch and plaque stages of MF (P7 = 0.001 and 0.004, respectively).

**Study limitations:**

This study is limited by the relatively small sample size of some clinical variants of mycosis fungoides, particularly advanced forms such as erythrodermic and tumor-stage MF. Further studies are needed to evaluate JAK1 and JAK3 expression following therapeutic interventions, including phototherapy and JAK inhibitors.

**Conclusions:**

JAK1 and JAK3 were significantly expressed in MF skin lesions in comparison to normal controls. JAK1 and JAK3 were more expressed in the tumor stage than patch and plaque stage MF, suggesting that JAK1 and JAK3 could play a crucial role in MF pathogenesis, progression, and prognosis.

## Introduction

Mycosis Fungoides (MF) is the most common subtype of cutaneous lymphoma, representing about 50% of all lymphomas arising primarily in the skin.^1^ It has an unclear cause, but various hypotheses are proposed. MF results from the malignant transformation of skin-resident effector memory T-cells.^2^, ^3^

Clinically, mycosis fungoides is usually associated with a prolonged indolent clinical course where the cases progress through three clinical phases: patch, plaque, and tumor stage.^1^ Recently, dermoscopy has been considered a confirmatory aid in MF diagnosis.^4^ Histopathologically, mycosis fungoides is characterized by an epidermotropic proliferation of small- to medium-sized pleomorphic cerebriform lymphocytes forming intraepidermal collections, so-called Pautrier microabscesses.^2^, ^5^

Janus Kinases (JAKs) are intracellular tyrosine kinases linked to intracellular domains of many cytokine receptors and are involved in signal transduction of various cytokine receptors. There are four JAK isoforms: JAK1, JAK2, JAK3 and Tyrosine Kinase-2 (TYK2). Different cytokine receptor families utilize specific JAK isoforms for signal transduction. Phosphorylation of JAK when a cytokine binds to its receptor leads to phosphorylation of Signal Transducer and Activator of Transcription (STAT) proteins, which dimerize and then translocate to the nucleus to directly regulate gene expression.^6^

Janus kinases have a critical role in the pathogenesis of some immune-mediated diseases, including rheumatoid arthritis, systemic lupus erythematosus and psoriatic arthritis.^7^ JAK inhibitors have shown efficacy in the treatment of dermatologic conditions such as atopic dermatitis, alopecia areata, psoriasis, and vitiligo.^8^

Deregulation of the JAK/STAT pathway signaling is an important feature of malignant T cells and has been implicated in the pathogenesis of a wide variety of T-cell malignancies, including adult T-cell leukemia/ lymphoma and early T-cell precursor acute lymphoblastic leukemia.^9^ The present study may help in understanding the role of JAK in the pathogenesis, prognosis, and severity of mycosis fungoides.

## Patients and methods

After approval of the research ethical committee (approval nº 33254/07/19), the present case-control study was conducted on 53 patients with different clinical variants of mycosis fungoides in addition to 53 healthy volunteers as controls. The study included newly diagnosed or previously diagnosed cases of mycosis fungoides who did not receive any systemic, topical, or phototherapy treatment for at least 3-months, who agreed to participate in the study and gave written consent. Pregnant or lactating females, patients with associated dermatological or systemic diseases were excluded. The patients were recruited from the local outpatient clinic of Dermatology and Venereology and the Clinical Oncology department.

All patients were subjected to complete history taking, including age and sex, full general examination to detect any associated systemic diseases, routine laboratory investigations, present history of illness, including onset, course, and duration of skin lesions, site of lesions, and associated symptoms: itching, pain and scaling.

### Clinical assessment of MF patients

Detailed dermatological examinations were done to determine the clinical variant, distribution, and the extent of mycosis fungoides and to assess the disease severity using the TNMB classification system.^10^ The body surface area involved in the patients was calculated using the patient's palm (the palm without fingers) to represent 0.5% body surface area.^11^ Clinical digital photographs were taken of the skin lesions of all patients using a digital camera Sony: cyber-shot DSC-WX 300 (20× optical zoom –18.2 megapixels). Imaging for staging of MF included abdominopelvic US, Neck, axillary, and inguinal US for lymph nodes assessment (if clinically involved LNs) and chest CT. The enlarged lymph nodes were excised and biopsied for histopathological Dutch grading of LNs in the Clinical Oncology Department.

### Histopathological assessment of MF patients

4 mm punch skin biopsy specimens were taken from the lesional skin of each patient to prepare paraffin-embedded blocks, then five tissue sections of 3‒4 μm thickness on glass slides. One section was stained with Hematoxylin and Eosin (H&E), and 4 sections were mounted on positively charged slides and stored at room temperature for immunohistochemical staining for CD3, CD4, JAK1, and JAK3.

### Hematoxylin and eosin staining

Was done for confirmation of clinical diagnosis and classification of classic MF into patch, plaque and tumor stage according to density of dermal lymphocytic infiltrate, with grading of the lymphocytic atypia in MF skin lesions, for epidermal and dermal lymphocytes, was based on examination of the all section to evaluate the number and size of the lymphocytes and were scored using the criteria of MF histopathological features proposed by Guitart et al.^12^ as follows:

Degree of lymphocytic atypia (for epidermal and dermal lymphocytes): 0: No atypia; 1: Mild atypia (small and intermediate cells); 2: Moderate atypia (small, intermediate plus large atypical cells); 3: Uniformly atypical or pleomorphic cells, many mitotic figures, few small round reactive lymphocytes. Lymphocytes are mostly intermediate and/or large in size.

Immunohistochemical staining of tissue sections using CD3 & CD4 immunostains for confirmation of the clinical diagnosis. The intensity of staining was qualitatively scored as negative (–), weak (+), moderate (++) and strong (+++) based on the pathologist's observations.

### Immunohistochemical staining of tissue sections using JAK monoclonal antibodies

JAK1 Monoclonal Antibody: Mouse synthetic peptide of JAK1 at AA range of 960‒1040. (Product code: CSB-MA919964, Cusabio Technology LLC).

JAK3 Antibody: synthetic peptide derived from human JAK3 around the non-phosphorylation site of Y785 (Product code: CSB-PA011293, Cusabio Technology LLC).

### Testing the antibody validation

To validate the antibodies’ performance and to determine if any of the antibodies show nonspecific binding, positive control samples were utilized, including breast carcinoma tissue for JAK1 and skin tissue with psoriasis for JAK3.

Slides were subjected to deparaffinization in xylene and rehydration through a graded alcohol series, then blocking the endogenous peroxidase activity by being placed in 3% hydrogen peroxide/methanol, followed by subsequent antigen unmasking. Incubation with the primary antibodies was performed for 30 minutes at room temperature using the following dilutions: 1:200 and 1:1200 for JAK1 & JAK3 antibodies, respectively. After washing with phosphate buffer saline, the slides were incubated for 30 min at room temperature with anti-mouse or anti-rabbit universal immunostaining IgG (biotin-conjugated secondary antibody) conjugated to a streptavidin peroxidase-labeled polymer (NeoMarkers Biotechnology). Reactions were developed with 3,3′-diamino benzidine-chromogen and counterstained with hematoxylin for 10 seconds, then washed with several changes of deionized water. Sections were dehydrated through alcohol 95%, then cleared through xylene. Excess xylene was then wiped off, and 1‒2 drops of permanent mounting media and a glass cover-slip were applied.^13^ Slides were mounted, and images were taken using the Leica DM500 microscope.

JAK1 expression was detected as homogenous brown stain in the nuclei of keratinocytes and lymphocytes in the epidermis and dermis respectively in addition to endothelial lining of the dermal blood vessels, hair follicles, eccrine glands and ducts, nerve cells, fibroblasts (Nuclear expression), while JAK3 expression was detected as homogenous brown stain in the cytoplasm of keratinocytes in the epidermis and lymphocytes in the dermis and similarly in the endothelial lining of the dermal blood vessels, hair follicles, eccrine glands and ducts, nerve cells and fibroblasts (cytoplasmic expression).

### Scoring of JAK1 & JAK3 expression in tissue biopsies

The scores were based on examination of the whole section in each biopsy, and immunostaining was scored under the Immunoreactivity Score (IRS).^14^ The IRS is the result of the multiplication of the ordinal scores for distribution and intensity of immunostaining. It combines two parameters: the percentage of positive cells and the intensity of staining. Percentage of Positive Cells (A): 0 = No positive cells, 1 = < 10% positive cells, 2 = 10%–50% positive cells, 3 = 51%–80% positive cells, 4 = > 80% positive cells. Intensity of Staining (B): 0 = No color reaction, 1 = Mild reaction, 2 = Moderate reaction, 3 = Intense reaction. IRS Score Calculation: IRS Score = A × B = 0–12. IRS Score Interpretation: 0–1 = Negative, 2–3 = Mild expression, 4–8 = Moderate expression, 9–12 = Strongly positive expression.

The quantitative evaluation of JAK1 and JAK3 expression was performed using the IRS score, with the primary focus of the scoring on epidermal and dermal lymphocytes. Other cell types, including keratinocytes, endothelial cells of blood vessels, eccrine glands, hair follicles, and nerve cells were noted descriptively but were not included in the main IRS scoring.

Real-time Quantitative Reverse Transcription Polymerase Chain Reaction (RT*-PCR)* for JAK1 and JAK3 after total RNA extraction from the paraffin-embedded tissue was done, and the methodology will be explained in detail in Part-2.

### Statistical analysis

Data were analyzed using the IBM SPSS software package version 20.0. (Armonk, NY: IBM Corp). Categorical data were represented as numbers and percentages. The Chi-Square test was applied to compare two groups. Alternatively, Fisher's Exact correction test was applied when more than 20% of the cells have expected count less than 5, Monte Carlo correction test was applied when more than 20% of the cells have expected count less than 5, Student *t*-test was used to compare two groups for normally distributed quantitative variables while ANOVA was used for comparing the four studied groups a. On the other hand, Mann Whitney test was used to compare two groups for non-normally distributed quantitative variables, and Kruskal Wallis test was used to compare different groups for non-normally distributed quantitative variables, followed by the Post Hoc test (Dunn's multiple comparisons test) for pairwise comparison. The Spearman coefficient was used to correlate between not normally distributed quantitative variables Pearson coefficient was used to correlate between two normally distributed quantitative variables. The significance of the obtained results was judged at the 5% level.

## Results

The present case-control study included 53 patients with MF and 53 healthy controls. The patients’ demographics and H&E findings, with Comparison between MF patients and Controls according to Age and Sex were illustrated in Table 1. The control samples showed normal epidermis, and only an occasional superficial dermal perivascular mononuclear infiltrate was detected in a few cases.

**Table 1:** Distribution of Mycosis fungoides patients according to clinical and histopathological data (n = 53).

Age of MF patients (years)	
Mean ± SD.	39.5 ± 17.7
Median (Min. – Max.)	44 (5 – 70)
**Sex of MF patients, n (%)**	
Male	18 (34.0%)
Female	35 (66.0%)
**Age of normal controls (years)**	
Mean ± SD.	42.6 ± 12.3
Median (Min. – Max.)	45 (18 – 65)
**Sex of normal controls**	
Male	15(28.3%)
Female	38(71.7%)
**Duration of lesions (in years)**	
< 5	34 (64.2%)
5 – 10	16 (30.2%)
> 10	3 (5.7%)
Mean ± SD.	4.82 ± 5.23
Median (Min. – Max.)	3 (0.08 – 25)
**Clinical type, n (%)**	
Classic MF (Patch stage)	10 (18.9%)
Classic MF (Plaque stage)	9 (17%)
Classic MF (Tumor stage)	3 (5.7%)
Hypopigmented MF	16 (30.2%)
Hyperpigmented MF	7 (13.2%)
Poikilodermatous MF	5 (9.4%)
Erythrodermic MF	3 (5.7%)
**Family History, n (%)**	
Negative	53 (100%)
Positive	0 (0%)
**Histopathological data**	**Nº of patients (%)**
Epidermotropism	51 (96.2%)
Dermal lymphocytes infiltrate	53 (100%)
Papillary dermal lymphocytes infiltrate	50 (94.3%)
Papillary and reticular dermal lymphocytes infiltrate	3 (5.7%)
**Degree of lymphocytic atypia**	
Mild (1)	31 (58.5%)
Moderate (2)	17 (32.1%)
Severe (3)	5 (9.4%)
Pautrier microabcess	9 (17%)
Epidermal atrophy	5 (9.4%)
Prominent vascularity	9 (17.0%)
Basal hyperpigmentation & dermal melanophages	12 (22.6%)

MF, Mycosis Fungoides.

Regarding the relation between clinical types of MF, histopathological data by H&E, and staging in MF patients, all MF patients had epidermotropism, except for two patients of tumor stage MF lacking epidermotropism. All clinical types of MF had papillary dermal lymphocytic infiltrate, while the 3 patients of tumor stage MF showed additional reticular dermal lymphocytic infiltrate. Regarding the degree of lymphocytic atypia in different MF clinical types, there was a statistically significant increase in the degree of lymphocytic atypia in the tumor stage MF than in other clinical types (p = 0.001). Pautrier microabcess was statistically significantly increased in plaque stage MF (77.8%) than other clinical types (p < 0.001). Epidermal atrophy was present in all patients of poikilodermatous MF. Prominent vascularity was detected in all patients of poikilodermatous and erythrodermic MF, and one patient (11.1%) of plaque stage MF, with a statistically significant increase in poikilodermatous and erythrodermic MF than other MF types (p < 0.001). Basal hyperpigmentation and melanophages were statistically significantly increased in patients with hyperpigmented and poikilodermatous MF than other MF types (p < 0.001), Table 2.

**Table 2:** Relation between clinical type with histopathological data and staging in mycosis fungoides patients (n = 53).

	Clinical type							χ^2^	**^MC^**p
	Classic MF (patch stage) (n = 10)	Classic MF (plaques) (n = 9)	Classic MF (tumors) (n = 3)	Hypopigmented MF (n = 16)	Hyperpigmented MF (n = 7)	Poikilodermatous MF (n = 5)	Erythrodermic MF (n = 3)		
**Histopathological data**									
Epidermotropism	10 (100%)	9 (100%)	1 (33.3%)	16 (100%)	7 (100%)	5 (100%)	3(100%)	12.071^a^	0.004^a^
**Dermal lymphocytes infiltrate**									
Papillary dermal lymphocytes infiltrate	10 (100%)	9 (100%)	0 (0%)	16 (100%)	7 (100%)	5 (100%)	3 (100.0%)	17.621^a^	<0.001^a^
Papillary and reticular dermal lymphocytes infiltrate	0 (0%)	0 (0%)	3 (100.0%)	0 (0%)	0 (0%)	0 (0%)	0 (0%)		
**Degree of lymphocytic atypia**									
Mild (1)	9 (90.0%)	3 (33.3%)	0 (0.0%)	11 (68.8%)	5 (71.4%)	3 (60.0%)	0 (0.0%)	25.228^a^	0.001^a^
Moderate (2)	1 (10.0%)	5 (55.6%)	0 (0.0%)	5 (31.3%)	2 (28.6%)	2 (40.0%)	2 (66.7%)		
Severe (3)	0 (0.0%)	1 (11.1%)	3 (100.0%)	0 (0.0%)	0 (0.0%)	0 (0.0%)	1 (33.3%)		
Pautrier microabcess	0 (0%)	7 (77.8%)	1 (33.3%)	0 (0%)	0 (0%)	0 (0%)	1 (33.3%)	24.536^a^	<0.001^a^
Epidermal atrophy	0 (0%)	0 (0%)	0 (0%)	0 (0%)	0 (0%)	5 (100%)	0 (0%)	24.417^a^	<0.001*
Prominent vascularity	0 (0%)	1 (11.1%)	0 (0%)	0 (0%)	0 (0%)	5 (100%)	3 (100%)	31.703^a^	<0.001^a^
Basal hyperpigmentation & dermal melanophages	0 (0%)	0 (0%)	0(0%)	0 (0%)	7 (100%)	5 (100%)	0 (0%)	42.990^a^	<0.001^a^
**Staging**									
Early stage MF	10 (100%)	8 (88.9%)	0 (0%)	16 (100%)	7 (100%)	5 (100%)	0 (0%)	26.227^a^	<0.001^a^
Late stage MF	0 (0%)	1 (11.1%)	3 (100%)	0 (0%)	0 (0%)	0 (0%)	3 (100%)		

χ^2^, Chi square test; ^MC^, Monte Carlo; p, p-value for comparing between the different categories; MF, Mycosis Fungoides.

a Statistically significant at p ≤ 0.05.

### Immunohistochemical examination

**Fig. 1:**
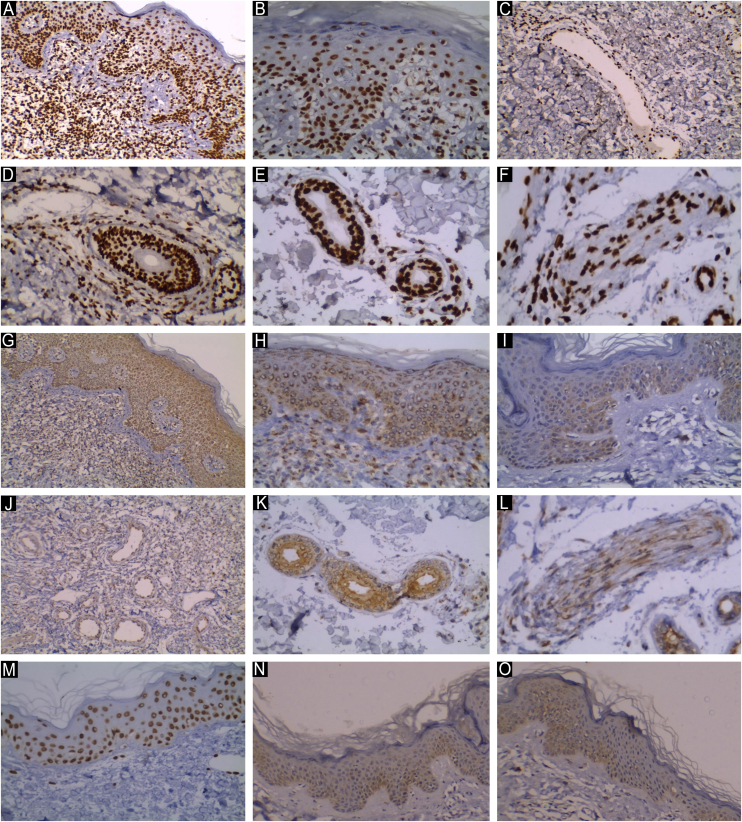
**JAK1 expression in MF patients**. (A) Strong nuclear expression of JAK1 in epidermal and dermal infiltrate, IRS = 12 (Immunostain for JAK1 × 200). (B) Moderate nuclear expression of JAK1 in epidermal and dermal infiltrate, IRS = 6 (Immunostain for JAK1 × 200). (C) Nuclear expression of JAK1 in endothelial cells in the wall of blood vessels perivascular lymphocytes. (D) Strong nuclear expression of JAk1 in the wall of hair follicles and perifollicular lymphocytic infiltrate. (E) Positive nuclear expression of JAK1 in eccrine glands and ducts. (F) Positive nuclear expression of JAK1 in nerve fibers. JAK3 expression in MF patients. (G) Strong cytoplasmic expression of JAK3 in epidermal and dermal infiltrate, IRS = 9 (Immunostain for JAK3 × 200). (H) Moderate cytoplasmic expression of JAK3 in epidermal and dermal infiltrate, IRS = 6 (Immunostain for JAK3 × 200). (I) Mild cytoplasmic expression of JAK3 in epidermal and dermal infiltrate, IRS = 3 (Immunostain for JAK3 × 200). (J) Numerous dilated blood vessels with positive staining of JAK3 in endothelial cells in the wall. (K) Positive cytoplasmic expression of JAK3 in eccrine gland and ducts. (L) Positive JAK3 in nerve fibers (cytoplasmic expression). JAK1 & JAK3 expression in normal controls. (M) Mild nuclear expression of JAK1, IRS = 2 (Immunostain for JAK1 × 200). (N) Mild cytoplasmic expression of JAK3, IRS = 3. Higher intensity of expression is noted in the basal cell layer of epidermis. (O) Note the weak positive staining in the endothelial lining of the dermal blood vessels.

In MF patients, JAK1 expression was detected as homogenous brown stain in the nuclei of keratinocytes and lymphocytes and in the epidermis and dermis respectively in addition to endothelial lining of the dermal blood vessels, hair follicles, eccrine glands and ducts, nerve cells, fibroblasts (Nuclear expression), while JAK3 expression was detected as homogenous brown stain in the cytoplasm of keratinocytes in the epidermis and lymphocytes in the dermis and similarly in the endothelial lining of the dermal blood vessels, hair follicles, eccrine glands and ducts, nerve cells and fibroblasts (cytoplasmic expression). The immuno-staining was scored under the Immunoreactivity Score (IRS), Fig. 1. Although JAK1 and JAK3 were detected in keratinocytes, vessels, eccrine glands, and other non-lymphoid cells, the IRS scoring primarily focused on epidermal and dermal lymphocytes, which are the main effector cells in MF. The expression in other cell types was described qualitatively to provide context on the tumor microenvironment.

The control skin samples showed nuclear JAK1 and cytoplasmic JAK3 expression. Higher intensity of JAK1 and JAK3 expression was noted in the basal cell layer in the epidermis. Weak positive staining of endothelial cells lining the wall of blood vessels and eccrine glands was noted (Fig. 1).

Janus kinase expression in MF patients compared to healthy controls using immunohistochemistry. Regarding the immunoreactivity score for JAK1, there was a statistically significant increase in MF patients than controls, with a p-value (< 0.001). On the other hand, the immunoreactivity score for JAK3 showed a statistically significant increase in MF patients than controls with p-value (< 0.001) (Table 3).

**Table 3:** Comparison between Mycosis fungoides patients and controls according to immunoreactivity score for JAK1 and JAK3 (all patients).

	MF patients (n = 53)	Controls (n = 53)	U	p
**IRS JAK1**				
Mean ± SD.	8.4 ± 2	2.5 ± 0.5	0.0^a^	<0.001^a^
Median (Min. – Max.)	9 (6 – 12)	2 (2 – 3)		
**IRS JAK3**				
Mean ± SD.	5.1 ± 2	3 ± 0	424.0^a^	<0.001^a^
Median (Min. – Max.)	6 (3 – 12)	3 (3 – 3)		

SD, Standard Deviation; U, Mann Whitney test; p, p-value for comparing between MF patients and controls; MF, Mycosis Fungoides; IRS, Immunoreactivity score.

a Statistically significant at p ≤ 0.05.

### JAK1 expression (IRS) in different MF clinical types using immunohistochemistry

**Fig. 2:**
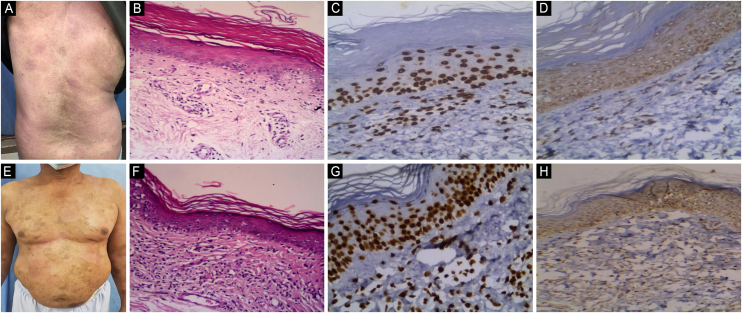
**JAK1 & JAK3 expression in Patch Stage Classic MF**. (A) Classic MF with multiple scaly atrophic erythematous patches on back. (B) Hematoxylin & eosin showing perivascular infiltrate of atypical lymphocytes in the dermis and basilar epidermotropism in the epidermis. (Hematoxylin & eosin, ×200). (C) Moderate nuclear expression of JAK1 in epidermal and dermal infiltrate. IRS = 6, intensity of staining = 3, Percentage of positive cells = 2, (Immunostain for JAK1 × 200). (D) Moderate cytoplasmic expression of JAK3 in epidermal and dermal infiltrate. IRS = 6 (Immunostain for JAK3 × 200). (E) Classic MF with disseminated erythematous, slightly atrophic scaly patches on trunk. (F) Hematoxylin and eosin showing basilar, pagetoid epidermotropism and dermal atypical lymphocytes (Hematoxylin & eosin, ×200). (G) Strong nuclear expression of JAK1 in epidermal and dermal infiltrate. IRS = 9 (Immunostain for JAK1 × 200). (H) Moderate cytoplasmic expression of JAK3 in epidermal and dermal infiltrate. IRS = 4 (Immunostain JAK3 × 200).

**Fig. 3:**
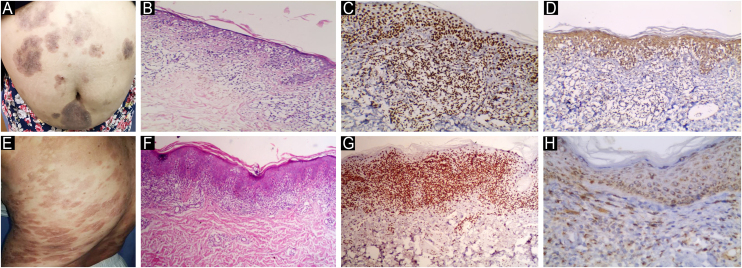
**JAK1 & JAK3 expression in Plaque Stage Classic MF**. (A) Classic MF with multiple erythematous brownish scaly plaques. (B) Hematoxylin & eosin showing extensive infiltrate of atypical lymphocytes in the upper dermis. Clear epidermotropism and pautrier microabcess are seen. (Hematoxylin & eosin, ×200). (C) Strong nuclear expression of JAK1 in epidermal and dermal infiltrate, IRS = 12 (Immunostain for JAK1 × 200). (D) Moderate cytoplasmic expression of JAK3 in epidermal and dermal infiltrate, IRS = 8 (Immunostain for JAK3 × 200). (E) Classic MF with multiple reddish brownish patches and plaques. (F) Hematoxylin and eosin showing plaque stage MF with heavy epidermotropism in the epidermis, and band like and perivascular infiltrate of atypical lymphocytes in the dermis. (Hematoxylin & eosin, ×40). (G) Strong nuclear expression of JAK1 in epidermal and dermal infiltrate, IRS = 9 (Immunostain for JAK1 × 200). (H) Moderate cytoplasmic expression of JAK3 in epidermal and dermal infiltrate, IRS = 6 (Immunostain for JAK3 × 200).

**Fig. 4:**
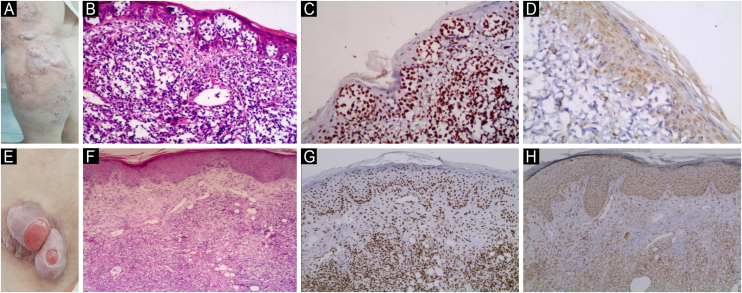
**JAK1 & JAK3 expression in Tumor Stage Classic MF**. (A) Tumor stage MF with multiple nodules, more than 1 cm in diameter. Some of these nodules arise on top of plaques of MF. (B) Hematoxylin & eosin showing dense infiltrate of atypical lymphocytes in the papillary and reticular dermis. Extensive epidermotropism and Pautrier microabcesses are seen in the epidermis (Hematoxylin & eosin, ×200). (C) Strong nuclear expression of JAK1 in epidermal atypical lymphocytes in Pautrier microabcesses and dermal infiltrate, IRS = 12 (Immunostain for JAK1 × 200). (D) Moderate cytoplasmic expression of JAK3 in epidermal and dermal infiltrate, IRS = 6 (Immunostain for JAK3 × 200). (E) Tumor stage of MF with large, ulcerated nodules on right side of chest. (F) Hematoxylin & eosin, showing heavy infiltrate of atypical lymphocytes in the papillary and reticular dermis with no epidermotropism (Hematoxylin & eosin, ×200). (G) Strong nuclear expression of JAK1 in epidermal and dermal infiltrate, IRS = 12. Note the weak staining in epidermis (no epidermotropism) (Immunostain for JAK1 × 200). (H) Strong cytoplasmic expression of JAK3 in epidermal and dermal infiltrate, IRS = 12 (Immunostain for JAK3 × 400).

**Fig. 5:**
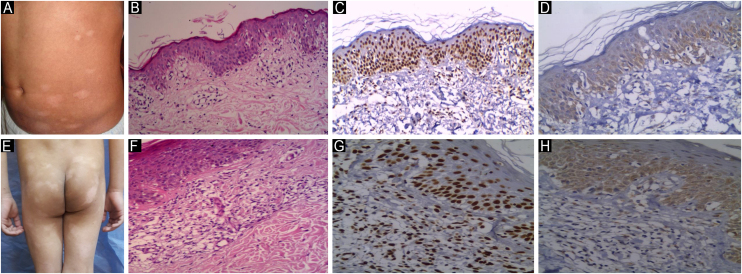
**JAK1 & JAK3 expression in Hypopigmented MF**. (A) Hypopigmented MF with ill-defined hypopigmented macules and patches. (B) Hematoxylin & eosin, showing epidermotropism and atypical lymphocytes in upper dermis. (Hematoxylin & eosin, ×200). (C) Strong nuclear expression of JAK1 in epidermal and dermal infiltrate, IRS = 9 (Immunostain for JAK1 × 200). (D) Mild cytoplasmic expression of JAK3 in epidermal infiltrate, IRS = 3 (Immunostain for JAK3 × 200). (E) Hypopigmented MF with hypopigmented slightly atrophic patches on back. (F) Hematoxylin & eosin showing band like infiltrate of atypical lymphocytes and epidermotropism (Hematoxylin & eosin, ×200). (G) Moderate nuclear expression of JAK1 in epidermal and dermal infiltrate, IRS = 6 (Immunostain for JAK1 × 200). (H) Moderate cytoplasmic expression of JAK3 in epidermal and dermal infiltrate, IRS = 6 (Immunostain for JAK3 × 200).

**Fig. 6:**
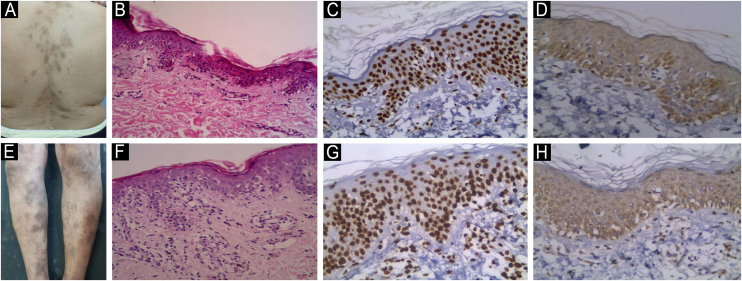
**JAK1 & JAK3 expression in Hyperpigmented MF**. (A) Hyperpigmented MF with scattered hyperpigmented patches with ill-defined irregular border, some of them show slight atrophy on trunk. (B) Hematoxylin & eosin showing epidermotropism of atypical lymphocytes, basilar hyperpigmentation and melanin incontinence in the dermis (Hematoxylin & eosin, ×200). (C) Moderate nuclear expression of JAK1 in epidermal cells and dermal infiltrate, IRS = 6 (Immunostain for JAK1, × 200). (D) Mild cytoplasmic expression of JAK3 in epidermal and dermal infiltrate, IRS = 3 (Immunostain for JAK3, × 200). (E) Hyperpigmented MF with brownish scaly patches on lower limbs. (F) Hematoxylin and eosin showing dermal infiltrate of atypical lymphocytes in the papillary dermis, perivascular lymphocytes and epidermotropism. Melanophages are present in the upper dermis (Hematoxylin & eosin, ×200). (G) Strong nuclear expression of JAK1 in epidermal and dermal infiltrate, IRS = 9 (Immunostain for JAK1, × 200). (H) Moderate cytoplasmic expression of JAK3 in epidermal and dermal infiltrate, IRS = 6 (Immunostain for JAK3, × 200).

**Fig. 7:**
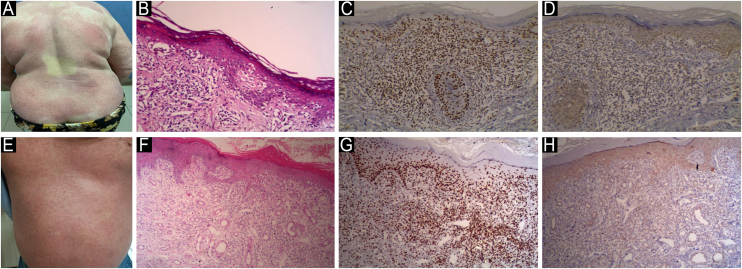
**JAK1 & JAK3 expression in Poikilodermatous MF**. (A) Poikilodermatous MF with pigmented scaly network and telangiectasia. (B) Hematoxylin & eosin, showing extensive epidermotropism of atypical lymphocytes in the epidermis. Numerous blood vessels and atypical lymphocytes are seen in the dermis (Hematoxylin & eosin, ×200). (C) Moderate nuclear expression of JAK1 in epidermal and dermal infiltrate, IRS = 6 (Immunostain for JAK1, × 200). (D) Moderate cytoplasmic expression of JAK3 in epidermal and dermal infiltrate, IRS = 4 (Immunostain for JAK3, × 200). **JAK1 & JAK3 expression in Erythrodermic MF**. (E) Erythrodermic MF with generalized scaling and erythema. (F) Hematoxylin and eosin showing basilar epidermotropism in the epidermis, infiltrate of atypical lymphocytes and numerous dilated blood vessels in the dermis (Hematoxylin & eosin, ×200). (G) Strong nuclear expression of JAK1 in epidermal and dermal infiltrate, IRS = 9 (Immunostain for JAK1, × 200). (H) Moderate cytoplasmic expression of JAK3 in epidermal and dermal infiltrate, IRS = 6 (Immunostain for JAK3, × 200).

There was a statistically significant increase in JAK1 IRS in the plaque stage than the patch stage (P1 = 0.001), and a statistically significant increase in the tumor stage than the patch stage (P2 = 0.001), with no significant difference between plaque and tumor stage (P3 = 0.313). When comparing patch, plaque, and tumor stage MF, there was a significant difference with higher expression of JAK1 in the tumor stage than in the patch and plaque stage MF (P7 = 0.001), (Table 4, Fig. 2, Fig. 3). JAK1 IRS was higher in tumor stage MF than hypopigmented MF with a statistically significant difference (P4 = 0.002). JAK1 IRS was higher in hyperpigmented MF than hypopigmented MF, with no statistically significant difference (P5 = 0.974). When comparing JAK1 IRS in hypopigmented, hyperpigmented, poikilodermatous, and erythrodermic MF, there was no statistically significant difference (P6 = 0.552). There were statistically significant differences regarding JAK1 expression between the different clinical types of MF (p = 0.001), (Table 4, Figs. 4‒7 ).

**Table 4:** Relation between JAK expression by immuno-histochemistry and clinical types in Mycosis fungoides patients (n = 53).

Clinical type	N	IRS JAK1		IRS JAK3		P8
		Mean ± SD	Median (Min. – Max.)	Mean ± SD	Median (Min. – Max.)	
Classic MF (Patch stage)	**10**	7.20 ± 1.55	6.0^b^, ^c^ (6.0 – 9.0)	4.50 ± 1.35	4.0^b^, ^c^ (3.0 – 6.0)	0.017^a^
Classic MF (Plaque stage)	**9**	10.33 ± 1.58	9.0 (9.0 – 12.0)	7.22 ± 1.56	8.0 (4.0 – 9.0)	0.007^a^
Classic MF (Tumor stage)	**3**	12.0 ± 0.0	12.0 (12.0 – 12.0)	8.67 ± 3.06	8.0 (6.0 – 12.0)	0.180
Hypopigmented MF	**16**	7.69 ± 1.54	9.0^b^, ^c^ (6.0 – 9.0)	3.75 ± 1.18	3.0^b^, ^c^ (3.0 – 6.0)	<0.001^a^
Hyperpigmented MF	**7**	7.71 ± 1.60	9.0 (6.0 – 9.0)	5.14 ± 1.46	6.0^b^ (3.0 – 6.0)	0.034^a^
Poikilodermatous MF	**5**	7.60 ± 1.52	8.0^b^, ^c^ (6.0 – 9.0)	4.60 ± 1.34	4.0^b^ (3.0 – 6.0)	0.039^a^
Erythrodermic MF	**3**	9.0 ± 0.0	9.0 (9.0 – 9.0)	5.33 ± 1.15	6.0 (4.0 – 6.0)	0.102
**H(p)**		23.366^a^ (0.001^a^)		25.199^a^ (<0.001^a^)		
**P1**		0.001^a^		0.004^a^		
**P2**		0.001^a^		0.021^a^		
**P3**		0.313		0.783		
**P4**		0.002^a^		0.001^a^		
**P5**		0.974		0.072		
**P6**		0.552		0.062		
**P7**		0.001^a^		0.004^a^		

H, H for Kruskal Wallis test, Pairwise comparison bet. Each 2 groups was done using Post Hoc Test (Dunn's for multiple comparisons test).

p, p-value for comparing between different clinical types; MF, Mycosis fungoides; IRS, Immunoreactivity Score.

P1: p-value for comparing between Patch stage and plaque stage.

P2: p-value for comparing between Patch stage and tumor stage.

P3: p-value for comparing between plaque stage and tumor stage.

P4: p-value for comparing between tumor stage and Hypopigmented MF.

P5: p-value for comparing between Hypopigmented MF and Hyperpigmented MF.

P6: p-value for comparing between hypopigmented, hyperpigmented, poikilodermatous and erythrodermic MF.

P7: p-value for comparing between Patch stage, plaque stage and tumor stage.

P8: p value for Wilcoxon signed ranks test for comparing between IRS JAK1 and IRS JAK3.

a Statistically significant at p ≤ 0.05.

b Significant with Classic MF (plaques).

c Significant with Classic MF (tumors).

### JAK3 expression in different MF clinical types using immunohistochemistry

There was a statistically significant increase of JAK3 IRS in the plaque stage than the patch stage (P1 = 0.004), and a statistically significant increase in the tumor stage than the patch stage (P2 = 0.021), and no significant difference between plaque and tumor stage (P3 = 0.783). When comparing patch, plaque, and tumor stage MF, there was a statistically significant difference with higher expression of JAK3 in the tumor stage than in the patch and plaque stage MF (P7 = 0.004), (Table 4, Fig. 2, Fig. 3). JAK3 IRS was higher in tumor stage MF than hypopigmented MF with a statistically significant difference (P4 = 0.001). JAK3 IRS was higher in hyperpigmented MF than hypopigmented MF, with no statistically significant difference (P5 = 0.072). When comparing JAK3 IRS in hypopigmented, hyperpigmented, poikilodermatous, and erythrodermic MF, there was no statistically significant difference (P6 = 0.062). There were statistically significant differences regarding JAK3 IRS level between the different clinical types of MF (p < 0.001) (Table 4, Figs. 4‒7).

### Comparison between JAK1 and JAK3 IRS in different MF clinical types

In the patch and plaque stage MF, JAK1 IRS showed a statistically significant increase than JAK3 IRS (p = 0.017, 0.007 respectively), while in tumor stage MF, JAK1 IRS was higher than JAK3 IRS with no statistically significant difference (p = 0.180). In hypopigmented, hyperpigmented, and poikilodermatous MF, JAK1 IRS showed a statistically significant increase compared to JAK3 IRS (p < 0.001, p = 0.034, p = 0.039, respectively), while in erythrodermic MF, JAK1 IRS was higher than JAK3 IRS with no statistically significant difference (p = 0.102), (Table 4).

### Relation between immunohistochemical expression of JAK1 and histopathological data in MF patients

The mean of JAK1 IRS in patients with epidermotropism was 8.22 ± 1.88, while in patients with absent epidermotropism (Two cases with tumor stage MF), the mean of JAK1 IRS was 12.0 ± 0.0, with a statistically significant increase of JAK1 IRS in patients with absent epidermotropism than in patients with epidermotropism (p = 0.017), (Table 5). The Mean of JAK1 IRS in patients with papillary and reticular dermal lymphocytes was 12.0 ± 0.0, while in patients with only papillary dermal lymphocytes, the mean of JAK1 IRS was 8.14 ± 1.8, and there was a statistically significant increase in patients with both reticular and papillary dermal infiltrate (p = 0.002). In MF patients with a mild degree of lymphocytic atypia, the mean of JAK1 IRS was 7.61 ± 1.5. In cases with moderate atypia, the mean of JAK1 IRS was 8.82 ± 1.98, while in patients with severe degree lymphocytic atypia, the mean of JAK1 IRS was 11.4 ± 1.34. There was a statistically significant increase in JAK1 IRS in patients with severe atypia than moderate and mild atypia (p = 0.001). In patients who showed Pautrier microabcesses in histopathology, the JAK1 IRS had a mean of 10.33 ± 1.58, in comparison to patients with absent Pautrier microabcesses, where the IRS of JAK1 had a mean of 7.95 ± 1.82, and there was a statistically significant increase in patients with Pautrier microabcesses than patients with absent microabcesses (p = 0.003). No significant relation was detected between JAK1 levels by immunohistochemistry in MF patients and the presence of epidermal atrophy, prominent vascularity, and the accentuated hyperpigmentation in the skin lesions of the patients (Table 5).

**Table 5:** Relation between JAK1 & JAK3 expression by immuno-histochemistry and histopathological data in Mycosis fungoides patients (n = 53).

Histopathological data	N	IRS JAK1		Test of sig.	p	IRS JAK3		Test of sig.	p
		Mean ± SD	Median (Min. – Max.)			Mean ± SD	Median (Min. – Max.)		
**Epidermotropism**									
Absent	**2**	12.0 ± 0.0	12.0 (12.0 – 12.0)	U = 5.0^a^	0.017^a^	10.0 ± 2.83	10.0 (8.0 – 12.0)	U = 3.50^a^	0.009^a^
Present	**51**	8.22 ± 1.88	9.0 (6.0 – 12.0)			4.92 ± 1.75	4.0 (3.0 – 9.0)		
**Dermal lymphocytes infiltrate**									
Papillary only	**50**	8.14 ± 1.82	9.0 (6.0 – 12.0)	U = 6.0^a^	0.002^a^	4.90 ± 1.76	4.0 (3.0 – 9.0)	U = 18.50^a^	0.023^a^
Papillary and reticular	**3**	12.0 ± 0.0	12.0 (12.0 – 12.0)			8.67 ± 3.06	8.0 (6.0 – 12.0)		
**Degree of lymphocytic atypia**									
Mild (1)	**31**	7.61 ± 1.5	9 (6 – 9)	H = 14.815^a^	0.001^a^	4.58 ± 1.52	4 (3 – 8)	H = 7.416^a^	0.025^a^
Moderate (2)	**17**	8.82 ± 1.98	9 (6 – 12)			5.29 ± 1.93	6 (3 – 8)		
Severe (3)	**5**	11.4 ± 1.34	12 (9 – 12)			7.80 ± 3.03	8 (4 – 12)		
**Pautrier microabcess**									
Absent	**44**	7.95 ± 1.82	9 (6 – 12)	U = 76.0^a^	0.003^a^	4.68 ± 1.88	4 (3 – 12)	U = 51.0^a^	<0.001^a^
Present	**9**	10.33 ± 1.58	9 (9 – 12)			7.22 ± 1.20	8 (6 – 9)		
**Epidermal atrophy**									
Absent	**48**	8.44 ± 2.02	9.0 (6.0 – 12.0)	U = 89.0	0.364	5.17 ± 2.08	6.0 (3.0 – 12.0)	U = 108.50	0.734
Present	**5**	7.60 ± 1.52	8.0 (6.0 – 9.0)			4.60 ± 1.34	4.0 (3.0 – 6.0)		
**Prominent vascularity**									
Absent	**44**	8.32 ± 2.03	9.0 (6.0 – 12.0)	U = 188.0	0.825	5.09 ± 2.11	5.0 (3.0 – 12.0)	U = 175.50	0.600
Present	**9**	8.56 ± 1.81	9.0 (6.0 – 12.0)			5.22 ± 1.56	6.0 (3.0 – 8.0)		
**Basal hyperpigmentation & dermal melanophages**									
Absent	**41**	8.56 ± 2.07	9.0 (6.0 – 12.0)	U = 186.50	0.164	5.17 ± 2.18	4.0 (3.0 – 12.0)	U = 242.50	0.938
Present	**12**	7.67 ± 1.50	8.50 (6.0 – 9.0)			4.92 ± 1.38	6.0(3.0 – 6.0)		

N, Number of patients; MF, Mycosis Fungoides; IRS, Immunoreactivity Score; U, Mann-Whitney test; H, H for Kruskal Wallis; p, p-value for comparing between different categories.

a Statistically significant at p ≤ 0.05.

### Relation between JAK3 expression by immunohistochemistry and histopathological data in MF patients

The mean of JAK3 IRS in patients with epidermotropism was 4.92 ± 1.75, while in patients with absent epidermotropism (Two patients with tumor stage MF), the mean of JAK3 IRS was 10.0 ± 2.83, with a statistically significant increase in patients with absent epidermotropism than patients with epidermotropism (p = 0.009). JAK3 IRS in patients with papillary and reticular dermal lymphocytes had a mean of 8.67 ± 3.06, compared to patients with papillary dermal lymphocytes only, JAK3 IRS had a mean of 4.90 ± 1.76, and there was a statistically significant increase in patients with both papillary and reticular dermal infiltrate (p = 0.023). In MF patients with a mild degree of lymphocytic atypia, the mean of JAK3 IRS was 4.58 ± 1.52. In patients with moderate atypia, the mean of JAK3 IRS was 5.29 ± 1.93. In patients with severe lymphocytic atypia, JAK3 IRS had a mean of 7.80 ± 3.03. There was a statistically significant increase of JAK3 IRS in patients with severe atypia compared to moderate and mild atypia (p = 0.025). In patients with Pautrier microabcesses, JAK3 IRS ranged from 6 to 9 with a mean of 7.22 ± 1.20, in comparison to the patients with absent Pautrier microabcesses where the IRS of JAK1 ranged from 3 to 12 with a mean of 4.68 ± 1.88, and there was statistically significant increase in patients with Pautrier microabcesses than patients with absent Pautrier microabcesses (p < 0.001). No significant difference was found in JAK3 IRS in MF patients with epidermal atrophy, prominent vascularity, or hyperpigmentation (Table 5).

### Relation between JAK1 and JAK3 expression and different stages of MF using immunohistochemistry

There was a statistically significant increase in the IRS of JAK1 than JAK3 IRS in the early stage (p < 0.001), and a statistically significant increase in JAK1 IRS than JAK3 IRS in late-stage MF (p = 0.027) (Table 6).

**Table 6:** Relation between JAK expression by immuno-histochemistry and staging in Mycosis fungoides patients (n = 53).

Staging	N	IRS JAK1		IRS JAK3		p
		Mean ± SD	Median (Min. – Max.)	Mean ± SD	Median (Min. – Max.)	
Early stage MF	**46**	8.0 ± 1.79	9.0 (6.0 – 12.0)	4.80 ± 1.76	4.0 (3.0 – 9.0)	<0.001^a^
Late stage MF	**7**	10.71 ± 1.60	12.0 (9.0 – 12.0)	7.14 ± 2.54	6.0 (4.0 – 12.0)	0.027^a^
**U (P1)**		51.0^a^ (0.002^a^)		70.50^a^ (0.015^a^)		

MF, Mycosis Fungoides; IRS, Immunoreactivity Score; U, Mann Whitney test; P1, p-value for comparing between different categories; p, p-value for Wilcoxon signed ranks test for comparing between IRS JAK1 and IRS JAK3.

a Statistically significant at p ≤ 0.05.

### Correlation between JAK1 and JAK3 expression in MF patients

A significant positive correlation was detected between JAK1 and JAK3 levels using immunohistochemistry in MF patients (*r* = 0.549 and p < 0.001, Table 7).

**Table 7:** Correlation between JAK1 and JAK3 regarding expression in Mycosis fungoides patients (n = 53).

JAK1 vs. JAK3	*r*_s_	p
IRS	0.549	<0.001^a^

*r*_s_, Spearman coefficient; IRS, Immunoreactivity Score.

a Statistically significant at p ≤ 0.05.

### Results of Real-time Quantitative Reverse Transcription Polymerase Chain Reaction (RT-PCR) of JAK1 and JAK3

Will be discussed in Part 2.

## Discussion

Mycosis fungoides is the most common variant of primary cutaneous T-cell lymphoma, accounting for over half of the cases. It originates from the peripheral epidermotropic T-cells, specifically memory T-cells (CD45RO+), which express T-cell receptor and CD4+ immunophenotype. It is evolving clinically from patches on sun-protected skin, through plaques, to tumorous nodules, with the chief histopathologic criteria being epidermotropism.^15^ Mycosis fungoides remains a challenge to diagnose, stage, and manage.^15^

Janus Kinases are a group of non-receptor intracellular tyrosine kinases that play a key role in the pathogenesis of various inflammatory skin disorders, oncogenesis, and disease progression in several cancers, particularly hematological malignancies. The mammalian JAK family, which contains three JAKs (JAK1–3) and TYK2, could be activated by the binding of an extracellular ligand to various transmembrane receptors, leading finally to upregulation of the transcription of various genes involved in cellular growth and hematopoiesis.^16^

JAK/STAT signaling plays a central role in mediating inflammation. Chronic inflammation is a critical hallmark of CTCL. Deregulated Janus Kinase signaling and activation of its STATs targets are common features of malignant T-cells. It appears to play an important role in cytokine expression, proliferation, and resistance to apoptosis in malignant T-cells and has been implicated in the pathogenesis of a wide variety of human cancers, including CTCL.^17^, ^18^, ^19^ The present study investigated the role of JAK1 and JAK3 in the pathogenesis, prognosis, and severity of mycosis fungoides.

The study included 53 patients with different clinical variants of MF, diagnosed clinically and confirmed by histopathology and cluster differentiation, in addition to 53 normal skin specimens as controls.

In this study, the immunohistochemical expression of JAK1 and JAK3 in MF was investigated and the JAK1 and JAK3 results were correlated with clinical and histopathological parameters. The present work demonstrated that immunohistochemical expression of JAK1 and JAK3 was significantly higher in MF patients than the healthy control group (p < 0.001 and p < 0.001 respectively). This could be explained by high JAK expression in cells with a high rate of proliferation and growth.^20^

Regarding JAK1 expression in other CTCL, consistent expression of activated JAK1 and JAK2 in primary Sézary syndrome samples and downregulation of JAK1 and JAK2 expression following the use of a pan-JAK inhibitor were demonstrated by McKenzie et al. 2012.^21^ However Eriksen et al. 2001^22^ observed that neither JAK1 nor TYK2 was phosphorylated or activated in leukemic Sézary cells.^22^

Regrading JAK3 expression in other CTCL, Eriksen et al. 2001^22^ observed the activation and constitutive phosphorylation of STAT3 and JAK3 in Sézary syndrome cells and the ability of the JAK inhibitor, tyrphostin AG490, to inhibit the growth of the leukemic Sézary cells. Their findings supported the view that the activation of JAK/STAT system in vivo might enhance the sensitivity of the tumor to local cytokines and thus increase tumor growth.^22^

JAK1 was previously reported to be positively expressed in different cutaneous disorders like vitiligo in perilesional and lesional skin, in addition to its expression in a group of common inflammatory skin diseases, including psoriasis, lichen planus, systemic lupus erythematosus and others.^23^ The expression was more pronounced in the dermal inflammatory cells than in the epidermal keratinocytes.^24^

Psoriasis is another T-cell-mediated disease that is characterized by accumulation of activated benign T-lymphocytes in the epidermis and dermis. JAK1 transmits the signals of IL6, IL22, IL23, and IL12, which are all upregulated and included in psoriasis pathogenesis. Therefore, JAKs has become a promising therapeutic target in different skin diseases, including psoriasis, atopic dermatitis, and alopecia areata, where selective modulation of the immune system can be useful.^16^, ^25^, ^26^

JAKs expression was also reported in some malignancies. Activating mutations of JAK1 and JAK3 were identified among adult T-cell leukemia/lymphoma and early T-cell precursor acute lymphoblastic leukemia.^9^ Additionally, Tang et al. 2019^27^ proved that JAK1 in the JAK protein family is closely associated with cancer development. The high expression of JAK1 in colon cancer tissues and its association to clinical staging, tumor depth, and lymph node metastasis suggests that the activation of JAK1 expression levels may be related to the occurrence of colon cancer, invasion, metastasis, and progression.^27^

In the current study, JAK1 showed nuclear expression in keratinocytes and epidermotropic lymphocytes in the epidermis and in lymphocytes in the dermis, endothelial lining of the dermal blood vessels, hair follicles, eccrine glands and ducts, nerve cells and muscles fibroblasts, in addition to high level of nuclear expression of JAK1 in basal cell layer in both normal skin controls and in MF tumor skin lesions. This could be explained by the findings of Zouein et al. 2011^20^ who concluded that the nuclear localization of JAKs (JAK1 and JAK2 in particular) in certain cells could be detected under conditions associated with high rates of cell growth where they can function as epigenetic regulators of gene expression and be of particular significance under physiological and pathological conditions of heightened cellular growth.^20^ Putting in consideration that the basal cell layer is the primary site of mitotically active cells in the epidermis that give rise to cells of the outer skin layers, and that the basal layer is characterized by the presence of epidermal stem cells which could progress and proliferate for skin renewal under normal conditions, and divide more rapidly with increase of the number of cycling cells under pathological conditions like skin wounding.^28^ The nuclear presence and high expression of JAK1 in epidermis and dermis of MF skin lesions and its association to high rate of cell growth, could suggest the role of JAK1 in MF pathogenesis and progression.

In the present study, JAK3 expression was cytoplasmic in the epidermal cells, dermal lymphocytes, hair follicles, sweat glands, muscles and nerve fibers. The original site of JAKs is cytoplasmic, where they could be stimulated by cytokine binding and followed by phosphorylation of STATs that translocate to the nucleus and control transcription of different genes that may be involved in cellular proliferation.^16^, ^29^ The high cytoplasmic JAK3 in this study could suggest the canonical pathway of JAK3 in MF pathogenesis.

Vadivel et al. 2021^30^ proved that JAK3 was expressed in the nuclear and cytoplasmic extracts of primary malignant T-cells and T-cell lines from patients with Sézary syndrome, using western blotting and immunofluorescence methods. Nuclear translocation of JAK3 was independent of its kinase activity, and JAK3 could interact with the nuclear protein POLR2A, the catalytic subunit of RNA Polymerase II, producing tyrosine phosphorylation of recombinant human Histone H3 by JAK3 in vitro, supporting the hypothesis that JAK3 may play a role in nuclear signaling in malignant T-cells.^30^ The presence of JAK3 in the nucleus of malignant T-cells suggests a non-canonical role of JAK3 in the proliferation and survival of malignant T-cells and their function in the CTCL pathogenesis.^30^

This study compared JAK1 and JAK3 expression in different MF clinical types. In the present work, the expression of JAK1 and JAK3 was more pronounced in tumor stage MF than plaque MF, patch MF, and other clinical subtypes of MF with statistically significant differences. Hypopigmented MF had the lowest level of JAK3. In all clinical types of MF, the expression of JAK1 was higher than the expression of JAK3.

In this study, being highly expressed in more advanced MF lesions as tumorous MF, JAK1, and JAK3, might be related to the severity of MF. The strong expression of JAK1 and JAK3 in tumor stage MF, which had a denser and deeper lymphocytic infiltrate than other MF clinical types and a higher degree of lymphocytic atypia than other MF types in this study, could be due to the high proliferative index and atypicality of the cells in this stage, hence expressed in cells with a high rate of proliferation.^20^, ^31^ In the same way, the low expression of JAKs in hypopigmented MF might be related to the low lymphocytic atypia in this MF clinical type observed by Jayasinghe et al. 2021.^32^

Regarding the correlation between immunohistochemical and histopathological findings in MF patients, the current study demonstrated a statistically significant higher level of JAK1 expression in patients that showed absent epidermoptropism, denser dermal lymphocytic infiltrate, and Pautrier microabcesses than patients with epidermotropism, papillary dermal infiltrate only and absent Pautrier microabcesses respectively. Also, in the current study, a statistically significant increase in JAK3 expression was demonstrated in patients with absent epidermoptropism, heavier dermal lymphocytes infiltrate, and patients with Pautrier microabcesses than in patients with epidermotropism, papillary dermal infiltrate only, and absent Pautrier microabcesses, respectively (p = 0.009, 0.023, < 0.001, respectively).

The JAK/STAT signal transduction pathway has been reported to be able to promote the expression of many growth factors, such as vascular endothelial growth factor A, insulin-like growth factor-1, and matrix metalloproteinase, and can activate angiogenesis at the tumor site, thus promoting cell proliferation and survival and inhibiting apoptosis.^33^, ^34^ In natural killer/T-cell lymphoma, JAK3 phosphorylates EZH2, the functional enzymatic component of the PRC2, converting it into a transcriptional activator, thereby augmenting expression of a series of target genes that are involved in malignant T-cell proliferation.^35^ In this way, the role of JAK1 and JAK3 expression in T-cell survival and infiltrate accumulation in MF skin lesions could be clarified.^34^, ^36^ This may give a rationale for the high expression of JAK1 and JAK3 in MF skin lesions with denser dermal T-lymphocytic infiltrate and cases with accumulated migrated T-cells in the epidermis in the form of Pautrier microabcesses.

In agreement with the present results, constitutive JAK3 activation induced aberrant proliferation and survival of CD8^+^ and CD4^+^ T-cells, leading to lymphoproliferative diseases in mice, in a murine bone marrow transplantation model. Interestingly, The JAK3-AV – induced T-cell lymphoproliferative disorder had a prominent cutaneous involvement. The histopathology of these skin lesions showed frequent alignment of highly atypical lymphoid cells with markedly irregular to convoluted and cerebriform nuclei along the dermal-epidermal junction, with the appearance of frequent intraepidermal atypical lymphocyte collections resembling Pautrier microabcesses, the characteristic histologic feature of MF.^37^ These cells were positively stained for CD3^+^, and for the chemokine receptor CCR10, which is associated with the homing of T-cells to epidermis and is highly expressed on neoplastic T-cells of cutaneous T-cell lymphomas, including MF.^37^, ^38^ This also could explain the high expression of JAK3 in skin lesions owing to Pautrier microabcesses and extensive dermal lymphocytes and the possible role of JAK3 in the occurrence of epidermotropism.

The current results observed a positive correlation between JAK1 and JAK3 level, and the degree of lymphocytic atypia, with a significant increase in JAK1 and JAK3 immunoreactivity score in patients with severe atypia than in patients with mild and moderate atypia using immunohistochemistry. The role of JAKs in cellular growth and cellular differentiation, particularly in the hyperproliferative states as malignancies, may be responsible for this finding.^39^ Janus Kinase contributes to the stimulation of cell proliferation in benign and malignant states.^40^ JAKs mutation were found to be associated with malignant transformation in different myeloproliferative disorders.^41^ This might explain the high level of JAKs in patients with severe atypia of lymphocytes.

In the present work, the histopathological results showed few cases with absent epidermotropism were of tumor stage MF. These cases showed extensive papillary and reticular dermal infiltrate and scored 3 in lymphocytic atypia. On the same side, Muñoz-González et al. 2017^31^ said that epidermotropism may be normally absent in the tumor stage of MF.^31^ The upregulation of JAK1 and JAK3 in this setting, despite absent epidermotropsim, could be attributed to the high rate of mitosis, atypicality and active proliferation in the tumor stage in these cases.^31^

Krejsgaard et al. 2006^42^ and Lauenborg et al. 2015^43^ observed that the aberrantly activated JAK3/STAT5 pathway could stimulate the expression of vascular endothelial growth factor and lymphotoxin α (formerly known as tumor necrosis factor-β), a protein known to be angiogenic and lymphangiogenic, in CTCL cells, including patients with MF, SS and anaplastic large cell lymphoma. Lymphotoxin α can increase the production of IL-6 in malignant cells, thus promoting angiogenesis, inducing endothelial proliferation and tube formation, and therefore promoting tumor growth, spread, and survival.^42^, ^43^ This highlighted the role of JAK3 in MF cells' survival and might explain the increased JAK3 in MF skin lesions having prominent vascularity, despite being insignificantly different in the present study.

In the current study, there was no significant correlation between JAK1 or JAK3 levels in MF patients and disease duration, age, and sex of the patients. This denotes that these factors may have no effect on JAK1 and JAK3 expression in MF patients.

The present study showed a statistically significant increase in JAK1 expression than JAK3 expression in the early stage and also a statistically significant increase in JAK1 than JAK3 expression in late-stage MF (p < 0.001, p = 0.027 respectively). These findings could indicate that JAK1 expression may contribute to both early and late-stage MF and that JAK3 may be more important in late-stage MF development, so JAK1 and JAK3 could have synergistic roles in the pathogenesis of MF, as well as in its progression. Additionally, JAK1 expression showed a significant increase over JAK3 in all MF clinical types, suggesting the greater importance of JAK1 in the pathogenesis of different MF variants.

Seif et al. 2017^44^ mentioned that the JAK/STAT pathway has a critical role in the fate of CD4+ T-helper cells, the typical immunophenotype in MF.^15^, ^44^ The role of JAKs in the T-helper differentiation is summarized as: TYK2, JAK1 and JAK2 are important for IL-12 signaling, resulting in Th1 cell differentiation, and interferon-induced JAK1 could also help in this setting, whereas JAK1 and JAK3 are important for IL-4 signal transduction, resulting in Th2 cell differentiation.^26^, ^45^ In early stage MF, the skin has increased expression of IFN-γ, IL-12, and IL-2, while the malignancy progression to late stage MF is characterized by a concomitant increase in the expression of other cytokines, such as IL-4, IL-5, IL-10, IL-13, IL15 and IL17.^46^, ^47^ IFN-γ also transmits signals through JAK1 and can phosphorylate it.^48^ These data highlighted the importance of JAK1 and JAK3 in MF early and late-stage pathogenesis.

In conclusion, by investigating the JAK1 and JAK3 expression in different MF clinical types, the study demonstrated that JAK1 and JAK3 could play a crucial role in MF pathogenesis, progression, and prognosis. JAK1 and JAK3 tissue expression could be markers of MF progression and staging and may act as therapeutic targets for inhibition in MF management.

## ORCID ID

Basma Mourad Mohamed Ali: 0000-0001-5820-5496

Mohamed Moustafa Shareef: 0000-0001-7826-9964

Lamia Elgarhy: 0000-0001-5277-4751

## Financial support

None declared.

## Authors' contributions

Heba Saed El-Amawy: Study conception, clinical data collection, manuscript writing.

Basma Mourad Mohamed Ali: Clinical supervision, critical revision of the manuscript.

Mohamed Moustafa Shareef: Pathological and immunohistochemical analysis.

Lamia Elgarhy: Supervision, editing, and final approval of the manuscript.

## Research data availability

The entire dataset supporting the results of this study was published in this article.

## Conflicts of interest

None declared.
